# Regional Reliability of Combining CT Angiography-source Image and Non-contrast CT in Acute Ischemic Stroke

**DOI:** 10.7150/ijms.101166

**Published:** 2024-10-07

**Authors:** Rongyuan Xu, Dawei Zhao, Ru Wen, Junxiang Qian, Chunzi Huang, Xiaojuan Deng

**Affiliations:** 1 Department of Radiology, the Third Affiliated Hospital of Chongqing Medical University, Chongqing 400042, China.; 2 Department of Neurosurgery and Key Laboratory of Neurotrauma, Southwest Hospital, Army Medical University (Third Military Medical University), Chongqing 400038, China.; 3 Department of Radiology, Southwest Hospital, Army Medical University (Third Military Medical University), Chongqing 400038, China.

**Keywords:** Acute ischemic stroke, Computed tomography angiography source image, Deep perforator vessel infarction, Large vessel trunk infarction

## Abstract

**Purpose:** CT angiography-source image (CTA-SI) can be used as an effective alternative to diffusion-weighted imaging (DWI) for identifying acute ischemic stroke (AIS). This study investigates the reliability of combining CTA-SI with non-contrast CT (NCCT) for AIS diagnosis, with a focus on how different brain areas affect diagnostic accuracy.

**Methods:** Patients with various subtypes of AIS who underwent NCCT, CTA, and DWI from January to December 2022 were included. Two experienced neuroradiologists analyzed ischemic core across NCCT, CTA-SI, and NCCT+CTA-SI models, evaluating interobserver reliability and lesion detection rate.

**Results:** A total of 304 patients (63% male, age 67.2 ± 11.9 years) with AIS were included. The distribution of stroke subtypes was as follows: 23% large vessel trunk infarction, 46% deep perforator vessel infarction, 9% superficial perforator vessel infarction, 5% watershed infarction, and 17% infratentorial infarction. The interobserver reliability was substantial in the three image models, especially the NCCT+CTA-SI model (all *p*<0.05). The NCCT+CTA-SI model demonstrated higher lesion detection rate than the NCCT (59.20% vs 48.7%, *p*<0.05) and CTA-SI model (59.2% vs 45.4%, *p*<0.05), particularly when detecting large vessel trunk infarction (82.90% vs 58.60%, *p*<0.05) and deep perforator vessel infarctions (64.80% vs 44.40%, *p*<0.05).

**Conclusions:** The NCCT+CTA-SI model may be a valuable tool for evaluating AIS when DWI is not feasible. Smaller hospitals might consider adopting this combination for improved stroke diagnosis, highlighting the need for careful evaluation of deep perforator vessel infarction when large vessel trunk infarction is not evident.

## Introduction

Acute ischemic stroke (AIS) is a medical emergency caused by decreased blood flow to the brain, affecting millions worldwide. In 2020, the global prevalence of all stroke subtypes was 68.16 million people 1. The management of AIS is highly time-sensitive; patients lose approximately 14 billion nerve connections and 1.9 million brain cells every minute during a stroke 2. Therefore, timely and accurate assessment is critical in guiding appropriate treatment.

Current imaging modalities, particularly non-contrast CT (NCCT) and MRI, play a crucial role in the initial management of AIS by providing essential diagnostic information for treatment planning 3. NCCT, often combined with CT angiography of the brain and neck within the first 6 hours of symptom onset, is used to rule out large vessel occlusion 4. However, NCCT has limitations, including poor sensitivity for detecting early acute ischemic changes, especially in minor strokes, due to its low spatial resolution 5, 6. This limitation can lead to delayed or missed diagnoses, negatively impacting patient outcomes.

MRI, and specifically diffusion-weighted imaging (DWI), offers superior spatial resolution and a high sensitivity of 97% for detecting ischemic lesions, making it an excellent tool for identifying brain ischemia in transient ischemic attacks or minor strokes before changes are visible on NCCT 7, 8. However, the availability of DWI is not uniform across all stroke centers, particularly in primary stroke centers that are often the first point of care 9. Furthermore, the use of MRI in acute stroke scenarios can be constrained by factors such as patient agitation and safety screening challenges, limiting its practicality in urgent settings 10.

CT angiography-source image (CTA-SI) has emerged as a promising alternative to DWI for early infarct detection and localization. CTA-SI offers greater sensitivity than NCCT in identifying ischemic changes and demonstrates a stronger correlation with final infarct volume, suggesting that it may more accurately reflect the ischemic core compared to NCCT 4, 11-14. However, CTA-SI's performance can be inconsistent, heavily dependent on the timing of the imaging, and it is less effective for detecting small vessel infarctions, such as those occurring in the posterior fossa 4. Additionally, its role in diagnosing watershed infarction (WI), which is often due to reduced perfusion, has not been thoroughly explored.

Given these limitations, this study aims to investigate the diagnostic value of combining CTA-SI with NCCT in predicting the ischemic core across various AIS subtypes, including large vessel trunk infarction (LTI), deep perforator vessel infarction (DPI), superficial perforator vessel infarction (SPI), WI, and infratentorial infarction (IFI). We hypothesize that the combination of these two imaging modalities will provide a more comprehensive and accurate assessment of the ischemic core, thereby enhancing diagnostic accuracy and improving patient outcomes. By examining different stroke subtypes, this study seeks to deepen our understanding of CTA-SI's role in stroke imaging and its potential as a valuable tool in stroke care.

## Material and methods

### Patients

**Figure 1:**
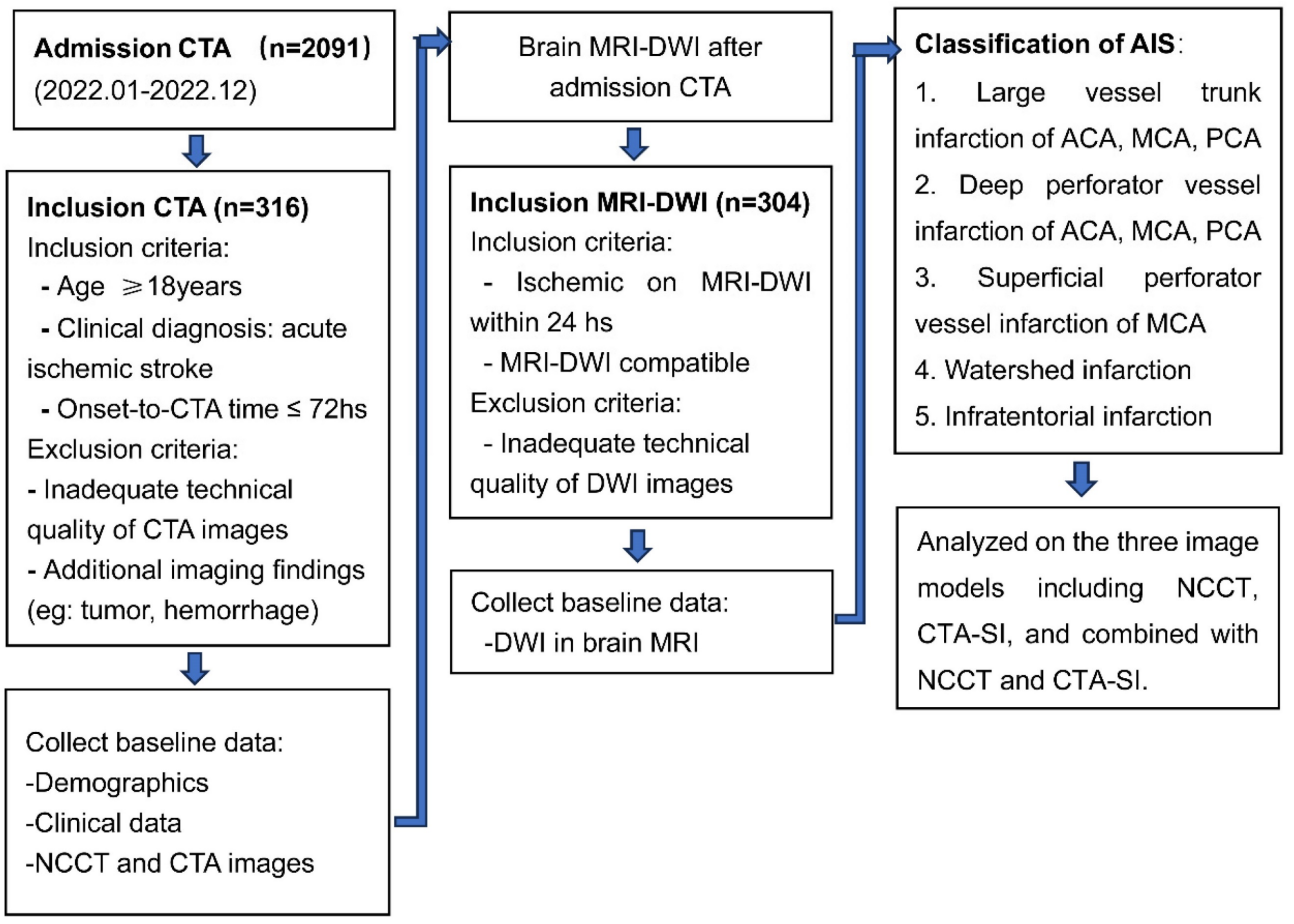
Study flowchart.

We retrospectively identified consecutive patients with acute ischemic stroke admitted to our hospital between January 1, 2022, and December 31, 2022. The inclusion criteria included the following: (1) presence of acute ischemic stroke; (2) age ≥ 18 years; (3) baseline imaging workup including NCCT, CTA, and DWI; (4) stroke onset or last known well ≤ 72 hours. Exclusion criteria were: (1) poor image quality; (2) the presence of tumor, hemorrhage, etc. that may influence functional outcome. The study flow chart is shown in **Figure 1**.

This study received approval from the ethics committee of the Third Affiliated Hospital of Chongqing Medical University (2022-73), and the requirement for patient consent was waived.

### Image protocol

Baseline CT was performed using a 256-slice spiral CT scanner (Revolution CT; GE Medical Systems, Chicago, IL, USA). CT protocol for patients with AIS included (1) NCCT (120 kVp, 100-350 auto-mAs, and contiguous 5mm axial sections ranging from the vertex to the skull base, reconstructed image layer thickness 2.5mm) and (2) CTA (manual switching of tube voltage 80-140kVp, automatic switching of tube current, detector width 80mm, pitch 0.992, adaptive statistical iterative reconstruction 60%, reconstructed image layer thickness 0.625mm). A 50mL non-ionic iodinated contrast (iopromide, Ultravist 350; Bayer Schering Pharma, Berlin, Germany) was administered at a flow rate of 5mL/s, followed by a 30mL saline chaser at the same rate. Follow-up DWI examinations were performed on a 3T scanner (Discovery 750W; GE Medical Systems, Chicago, IL, USA), TR 4347 ms, TE minimum, b values 0 and 1000 s/mm^2^, and slice thickness 6mm.

### Data collection and imaging analysis

**Figure 2:**
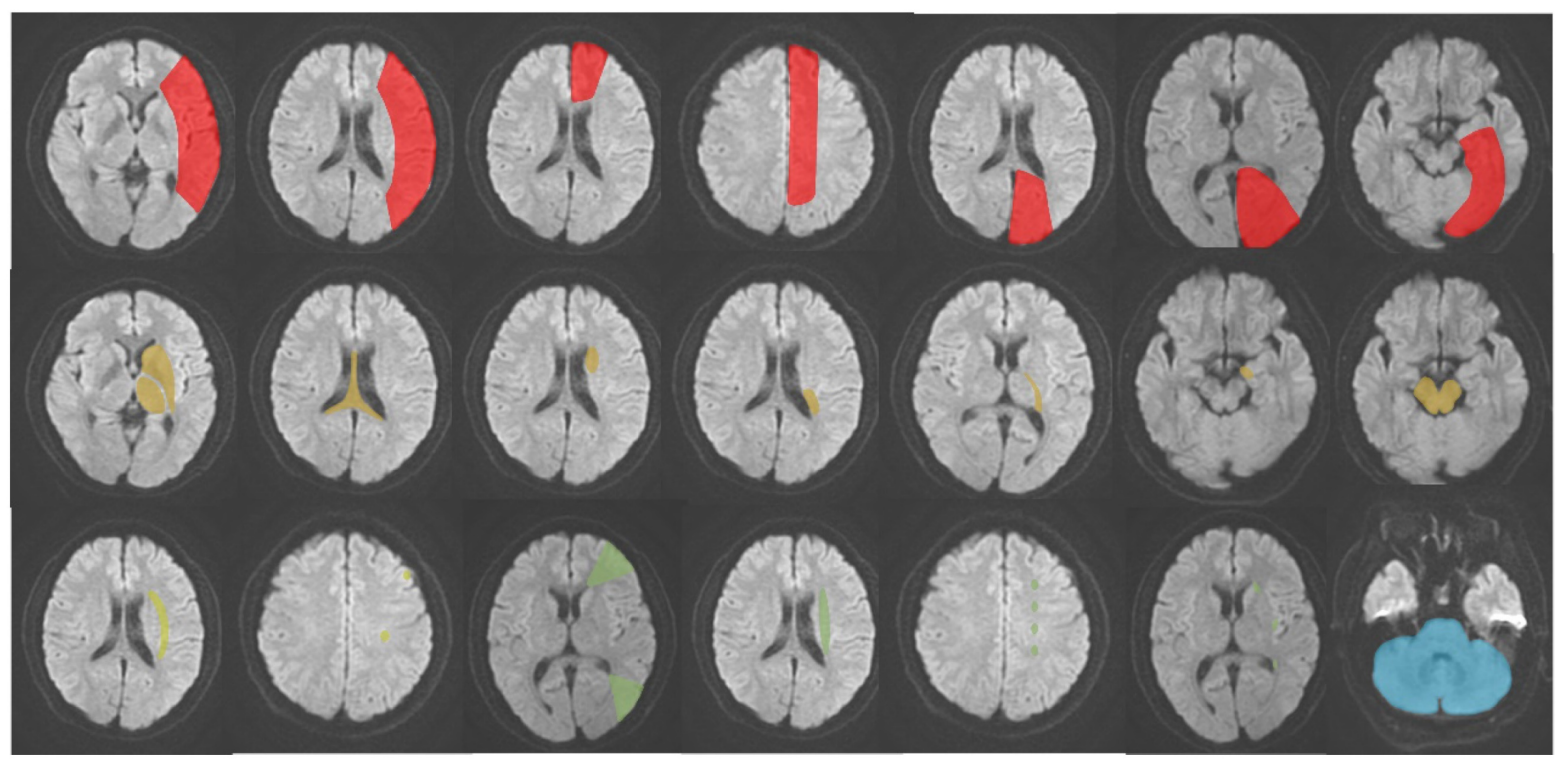
** The schematic diagram of AIS classification.** Red: large vessel trunk infarction; yellow: deep perforator vessel infarction; light yellow (lime): superficial perforator vessel infarction; green: watershed infarction; blue: infratentorial infarction.

AIS was classified into five subtypes based on its occurrence in different locations: (1) LTI of anterior cerebral artery (ACA), middle cerebral artery (MCA), and posterior cerebral artery (PCA); (2) DPI of ACA, MCA and PCA; (3) SPI of MCA; (4) WI; (5) IFI 14-17. The schematic diagram of the classification is presented in **Figure 2**. Hyperattenuating on DWI and hypoattenuating on ADC were defined as an ischemic core.

Three diagnostic models were developed, including NCCT, CTA-SI, and combined with NCCT and CTA-SI. Images were analyzed by 2 stroke neuroradiologists experienced in interpreting NCCT and CTA-SI in AIS (reader 1 with 17 years of experience, and reader 2 with 9 years of experience) who were blind to clinical information except for the side of stroke involvement. Before the start of the study, both readers underwent a joint training session on schematic diagrams of classifications of AIS to harmonize image interpretation. Readers then reviewed the NCCT image with a 2.5 mm slice thickness and CTA-SI with a 0.625 mm slice thickness on a large high-resolution monitor. First, they adjusted the angle to normal; the window and level were adjusted individually to allow maximum contrast produced by small attenuation differences between normal and ischemic tissue. Regions of relatively hypoattenuation on NCCT and diminished contrast enhancement on CTA-SI were considered abnormal. The two neuroradiologists randomly interpreted three image models at separate sessions 2-3 weeks apart.

### Statistical analysis

Statistical analysis was performed using IBM SPSS version 26 software (IBM SPSS Statistics, Armonk, NY). A normality test was performed on the measurement data. The data with normal distribution were expressed as mean±SD. Continuous nonnormal data were described as median [interquartile range (IQR)]. Enumeration data were expressed by the frequency and composition ratio (%). (1) The Kappa consistency test was used to analyze the diagnostic consistency between the two radiologists. Inter-observer reliability of lesion detection rate among the three models was determined by Cohen's Kappa, where the power of the kappa value was interpreted as poor (k=0.00), slight (k=0.00-0.20), fair (k=0.21-0.40), moderate (k=0.41-0.60), substantial (k=0.61-0.80) and excellent (k>0.81) agreement. (2) The chi-square test and multiple comparisons were used to compare the differences in the lesion detection rate of the three image models (NCCT, CTA-SI, NCCT+CTA-SI) in subtypes of AIS. *p*<0.05 was considered statistically significant.

## Results

### Patient demographic and baseline imaging characteristics

Among the 2091 patients with acute ischemic stroke who underwent CTA, 304 patients with documented infarction in DWI were identified. There were 193 (63%) males with a mean age of 67.2 years ± 11.9 years. The median time from CTA to DWI was 18 hours (IQR 9-22 hours). There were 70 (23%) patients with LTI; 142 (46%) with DPI; 26 (9%) patients had SPI; 14 (5%) patients had WI, and 52 (17%) patients had IFI. The baseline characteristics of the patients are shown in **Table 1**.

When detecting LTI, the interobserver reliability was substantial in the three image models (all p<0.05). For DPI, the interobserver reliability was substantial when the NCCT+CTA-SI model was used (p<0.05) but moderate for NCCT or CTA-SI models (p<0.05).

For WI, the interobserver reliability was excellent for all three models (p<0.05), while for IFI, the interobserver reliability was substantial for the NCCT+CTA-SI model (p<0.05).

### Interobserver reliability of lesion detection rate on NCCT, CTA-SI, and NCCT+CTA-SI in detecting different AIS subtypes

The interobserver reliability was substantial in the three image models, especially the NCCT+CTA-SI model (all p<0.05). For SPI, there was fair consistency among observers when using the NCCT+CTA-SI model, while interobserver consistency was substantial and excellent when detecting other AIS subtypes (**Table 2**).

### Diagnostic accuracy of three image models across different area subtypes of AIS

**Figure 3:**
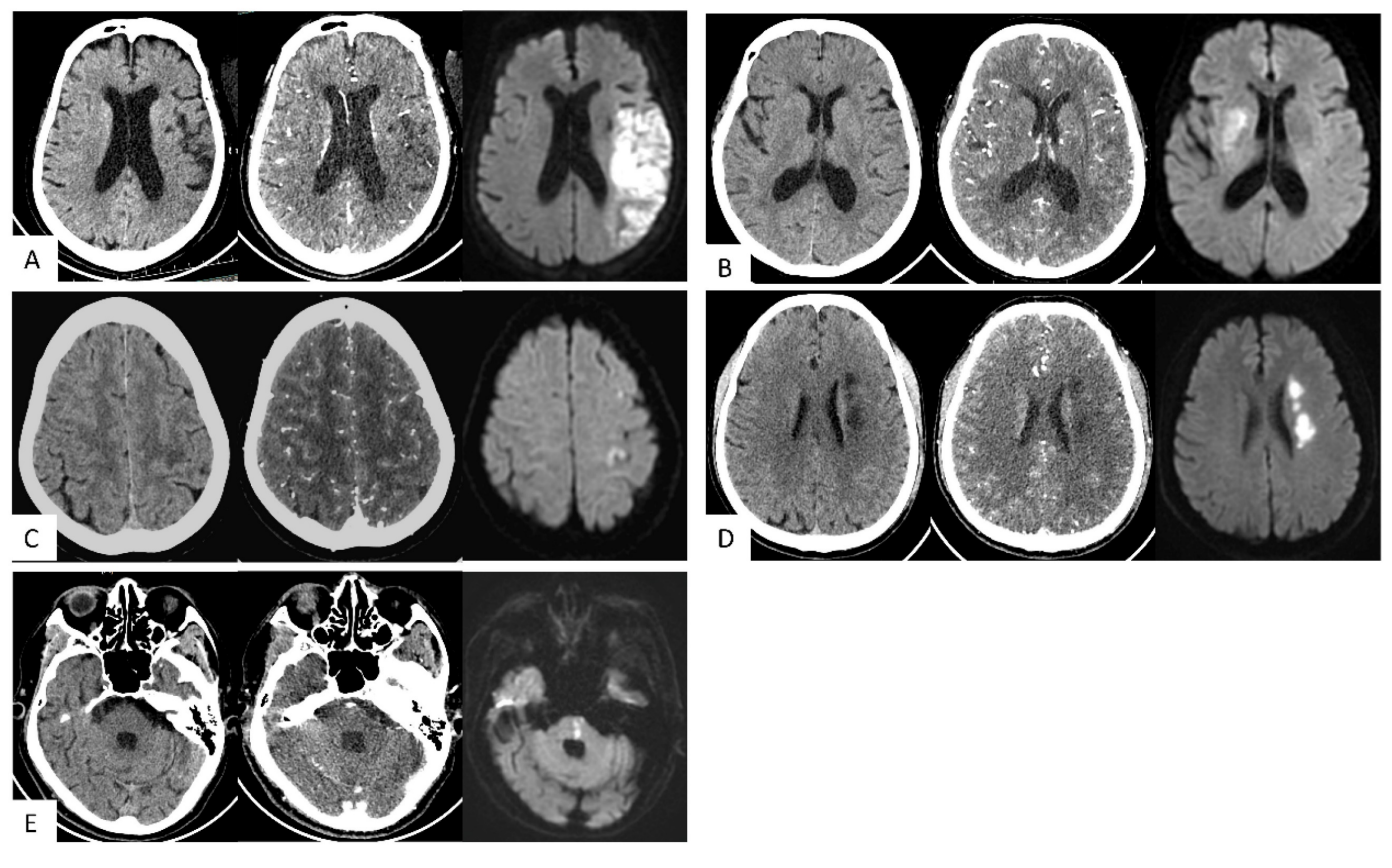
**Typical cases. (A) Large vessel trunk infarction (LTI).** An 85-year-old female; onset time 3+ hours. NCCT showed no obvious abnormalities. CT angiography source images (CTA-SI) revealed a large, slightly low attenuation in the left frontal and parietal lobes. The combination of CTA-SI and NCCT depicted left frontal and parietal lobe ischemic stroke. Diffusion-weighted imaging (DWI) showed a large, diffuse, restricted area in the left frontal and parietal lobes. **(B) Deep perforator vessel infarction (DPI).** A 67-year-old female; onset time 23 hours. NCCT showed no obvious abnormalities. CTA-SI revealed a slightly low attenuation in the right basal ganglia. The combination of CTA-SI and NCCT depicted right basal ganglia ischemic stroke. DWI showed a patchy diffuse restricted area in the right basal ganglia. **(C) Superficial perforator vessel infarction (SPI).** A 67-year-old female; onset time 24 hours. NCCT showed a slightly low attenuation in the subcortical region of the left parietal lobe. CTA-SI showed no obvious abnormalities. The combination of CTA-SI and NCCT did not indicate the infarct focus. DWI showed a patchy and punctate restricted area in the subcortical region of the left frontal and parietal lobe. **(D) Watershed infarction (WI).** A 61-year-old male; onset time 3+ hours. NCCT and CTA-SI demonstrated chainlike low attenuation in the left paraventricular region, and there were no signs of old lesions in the surrounding area. DWI showed a chainlike restricted area in the left paraventricular region. **(E) Infratentorial infarction (IF).** A 78-year-old male; onset time 1+ hours. NCCT and CTA-SI showed no obvious abnormalities in the posterior fossa. Beam hardening artifacts were shown on CTA-SI. DWI showed patchy restricted area in the pons which located in the posterior fossa.

NCCT+CTA-SI showed superior lesion detection rate compared to NCCT or CTA-SI models (all p<0.05). For LTI, the lesion detection rate of NCCT+CTA-SI was higher than NCCT (p<0.05); for DPI, the lesion detection rate of NCCT+CTA-SI was higher than the CTA-SI model (p<0.05). For the other three subtypes, no statistical significance was observed among the 3 models (**Table 3**). Typical case images are shown in **Figure 3A-E**.

## Discussion

Our data suggested that the combination of NCCT and CTA-SI has a relatively high detection rate for evaluating the final infarct volume, especially for detecting LTI and DPI, compared to NCCT or CTA-SI alone. NCCT+CTA-SI may be a helpful image model for evaluating AIS patients when MRI is not feasible.

The integration of NCCT and CTA-SI capitalizes on the respective strengths of both techniques. NCCT provides detailed anatomical visualization and serves as a reference for identifying ischemic changes 3, whereas CTA-SI offers valuable vascular information, facilitating the identification of ischemic tissue 4. The synthesis of anatomical and hemodynamic data from these imaging modalities suggests that the combined NCCT+CTA-SI protocol could potentially yield a more comprehensive and precise evaluation of the ischemic core, thereby minimizing the likelihood of both overestimation and underestimation of the affected region. The empirical evidence from our study corroborates this hypothesis, indicating that the NCCT+CTA-SI approach significantly improves lesion detection rates and enhances interobserver agreement when compared with the application of NCCT or CTA-SI in isolation.

Numerous studies 2-4 have suggested that CTA-SI can improve the diagnostic rate of acute cerebral infarction, which is consistent with the conclusion of this study. However, most of the analyses of CTA source images were performed in evaluating LTI, including diagnosis of acute large vessel occlusion 5, localization diagnosis of infarcts 6, prediction of infarct volume 7, and prognosis of clinical outcomes 8, while only a few reported on perforator infarction.

Lacunar infarcts are small (2 to 15 mm in diameter) infarcts caused by occlusion of a single penetrating branch of a large cerebral artery. They account for approximately 25% of acute ischemic strokes 9. Some studies have shown that a combination of NCCT and CTA can improve the detection of lacunar infarcts, yet the exact sensitivity and sensibility remain debatable 10-12.

Some studies have utilized the Alberta Stroke Program Early Computed Tomography Score (ASPECTS) to analyze CTA-SI 13. However, it should be noted that although ASPECTS included the infarcted area of perforating vessels, the score difference did not directly indicate the difference in the infarcted area. Accurately identifying the infarcted area is crucial in clinical practice for AIS, as it helps determine the responsible vessels. Our findings suggest that the combination of NCCT and CTA-SI not only enhances the detection of occlusion in major blood vessels but also facilitates the recognition of DPI, which accounts for nearly half of our stroke cases and could be shown in the basal ganglia and thalamus.

The superficial perforating arteries descend towards the upper part of the lateral ventricle and supply the centrum ovale. Embolic pathogenesis has a significant role in developing SPI. Studies have found that emboli small enough to lodge in the white matter medullary artery result in small infarcts 14. Also, spotty cortical lesions are frequently associated with SPI, and the infarcts tend to have a circular or oval morphology 14. This study found that neither NCCT nor CTA-SI is sensitive enough to detect SPI due to its small size and location in the cortex and subcortical layer, which could only be shown on other image models 14, 18.

Watershed infarcts are ischemic strokes located in vulnerable border zones between brain tissue supplied by the anterior, posterior, and middle cerebral arteries in the distal junction between two non-anastomotic arterial territories. Previous studies 16, 17 have suggested that these infarcts are caused by cerebral hypoperfusion due to intracranial artery occlusive lesions and arterial embolism in unstable atheroma plaque. WI tends to localize in the paraventricular region, appear chainlike or sausage-like, and are significantly larger than SPI 19. They can also present as triangular, cortical, cortico-subcortical, or periventricular shape lesions. Paraventricular chainlike lesions are a distinct type of deep cerebral infarct that can be morphologically distinguished by neuroimaging. Therefore, NCCT alone or combined with CTA-SI can be useful for diagnosis when there is no previous interference from infarcted lesions. In this study, the sensitivity of WI on the three image models was higher than that of SPI. However, diagnosis becomes challenging when the patient has previous interference from old lesions, such as softening or old hemorrhage, and triangular lesions in cortical or cortico-subcortical regions. Thus, radiologists should promptly recognize this type of stroke on the image in the shortest possible time.

Diagnosing acute posterior fossa infarct can be challenging due to the overlapping clinical signs and symptoms with supratentorial infarction. Although NCCT and CTA-SI can assist in the diagnosis, their limited sensitivity poses a significant challenge. Previous studies 20, 21 have reported a sensitivity range of only 15.38% to 41.8% on NCCT. While CTA-SI is a superior tool for visualizing ischemic, the sensitivity for posterior fossa infarct is only 32.2% on the combined model of NCCT and CTA-SI, which is consistent with our studies. The main reasons for the limited sensitivity are the radiologic particularities of the posterior fossa, such as beam hardening artifacts, especially in the brainstem, and artifacts caused by spiral scanning.

The study also found moderate to poor interobserver reliability in detecting lesions in AIS subtypes such as SPI and IFI. Contributing factors include imaging limitations, such as NCCT's low sensitivity for small lesions in subcortical and infratentorial regions, and challenges with CTA-SI in detecting atypical infarcts. Additional factors include variability in lesion characteristics, differences in observer experience, image quality issues, and the inherent subjectivity in interpretation.

This study has several limitations. First, as a retrospective, single-center study, the generalizability of the findings may be limited. Second, the analysis of certain infarct subtypes requires further investigation for more definitive conclusions. The successful application of the combined NCCT+CTA-SI model also depends on high-quality CT images and specialized radiologist training in stroke imaging. Future research should focus on multicenter, large-sample, and prospective studies to validate these findings and enhance their applicability in diverse clinical settings.

## Conclusions

In conclusion, the combination of NCCT and CTA-SI offers a higher detection rate for final infarct, particularly in LTI and DPI, compared to using NCCT or CTA-SI alone. This improved diagnostic sensitivity can enhance clinical decision-making in acute settings where rapid and accurate diagnosis is critical, especially in facilities without access to MRI. The NCCT+CTA-SI model supports timely management of AIS patients, aiding early and accurate identification of ischemic strokes to improve patient outcomes.

## Figures and Tables

**Table 1:** Patient demographic and baseline imaging characteristics

Variable	Cohort (n= 304)
Age, y; mean (±SD)	67.2 (±11.9)
Male, No. (%)	193 (63%)
Time from CTA imaging to DWI imaging, hs; median (IQR)	18 (9-22)
**Subtypes of Acute Ischemic Stroke**	
LTI, No. (%)	70 (23%)
DPI, No. (%)	142 (46%)
SPI, No. (%)	26 (9%)
WI, No. (%)	14 (5%)
IFI, No. (%)	52 (17%)

**Abbreviations:** CTA, CT angiography; DWI, diffusion-weighted imaging; IQR, interquartile range; LTI, large vessel trunk infarction; DPI, deep perforator vessel infarction; SPI, superficial perforator vessel infarction; WI, watershed infarction; IFI, infratentorial infarction

**Table 2:** Interobserver reliability of lesion detection rate on NCCT, CTA-SI, and NCCT+CTA-SI in subtypes of AIS

	NCCT			CTA-SI			NCCT+CTA-SI	
AIS subtypes	Kappa value 95% CI (lower, upper)	p		Kappa value 95% CI (lower, upper)	p		Kappa value 95% CI (lower, upper)	p
Total	0.626 (0.540-0.712)	0		0.659 (0.575-0.743)	0		0.734 (0.658-0.81)	0
LTI	0.657 (0.483-0.831)	0		0.757 (0.573-0.941)	0		0.757 (0.555-0.959)	0
DPI	0.56 (0.433-0.687)	0		0.553 (0.416-0.69)	0		0.646 (0.519-0.773)	0
SPI	1 (1-1)	0.038		1 (1-1)	0		0.339 (-0.235-0.913)	0.076
WI	0.837 (0.533-1.141)	0.005		1 (1-1)	0		0.837 (0.533-1.141)	0.001
IFI	0.505 (0.262-0.748)	0		0.349 (0.077-0.621)	0.01		0.718 (0.532-0.904)	0

**Abbreviations:** NCCT, non-contrast CT; CTA-SI, CT angiography-source image; NCCT+CTA-SI, combined with NCCT and CTA-SI; 95% CI, 95% confidence interval; LTI, large vessel trunk infarction; DPI, deep perforator vessel infarction; SPI, superficial perforator vessel infarction; WI, watershed infarction; IFI, infratentorial infarction

**Table 3:** Lesion detection rate of three image models across different area subtypes

Subtypes of Acute Ischemic Stroke	Accuracy			p-value
	NCCT (A)	CTA-SI (B)	NCCT+CTA-SI (C)	
Total (n=304)	48.70%	45.40%	59.20%	A vs. C p<0.05 B vs. C p<0.05
LTI (n=70)	58.60%	77.10%	82.90%	A vs. C p<0.05
DPI (n=142)	55.60%	44.40%	64.80%	B vs. C p<0.05
SPI (n=26)	15.40%	3.80%	11.50%	NS
WI (n=14)	35.70%	21.40%	28.60%	NS
IFI (n=52)	36.50%	32.70%	44.20%	NS

**Abbreviations:** NCCT, non-contrast CT; CTA-SI, CT angiography-source image; NCCT+CTA-SI, combined with NCCT and CTA-SI; 95% CI, 95% confidence interval; LTI, large vessel trunk infarction; DPI, deep perforator vessel infarction; SPI, superficial perforator vessel infarction; WI, watershed infarction; IFI, infratentorial infarction; numbers given for accuracy are percentages; NS, not significant

## Abbreviations

ACA: Anterior cerebral artery
AIS: Acute ischemic stroke
ASPECTS: Alberta Stroke Program Early Computed Tomography Score
CTA: CT angiography
CTA-SI: CT angiography-source image
DPI: Deep perforator vessel infarction
DWI: Diffusion-weighted imaging
IFI: Infratentorial infarction
IQR: Interquartile range
LTI: Large vessel trunk infarction
MCA: Middle cerebral artery
NCCT: Non-contrast CT
PCA: Posterior cerebral artery
SPI: Superficial perforator vessel infarction
WI: Watershed infarction
